# Annealing of ion tracks in apatite under pressure characterized *in situ* by small angle x-ray scattering

**DOI:** 10.1038/s41598-020-57600-y

**Published:** 2020-01-28

**Authors:** Daniel Schauries, Boshra Afra, Pablo Mota-Santiago, Christina Trautmann, Maik Lang, Rodney C. Ewing, Nigel Kirby, Patrick Kluth

**Affiliations:** 1 Department of Electronic Materials Engineering, Research School of Physics, Australian National University, Canberra, ACT 2601 Australia; 2 GSI Helmholtz Centre for Heavy Ion Research, Planckstrasse 1, 64291 Darmstadt, Germany; 3 Technische Universität Darmstadt, 64287 Darmstadt, Germany; 4 Department of Nuclear Engineering, University of Tennessee, Knoxville, Tennessee 37996 USA; 5 Department of Geological Sciences, Stanford University, Stanford, CA 94305-2115 USA; 6 Australian Synchrotron, 800 Blackburn Road, Clayton, VIC 3168 Australia

**Keywords:** Geophysics, Characterization and analytical techniques

## Abstract

Fission track thermochronology is routinely used to investigate the thermal history of sedimentary basins, as well as tectonic uplift and denudation rates. While the effect of temperature on fission track annealing has been studied extensively to calibrate the application of the technique, the effect of pressure during annealing is generally considered to be negligible. However, a previous study suggested elevated pressure results in a significantly different annealing behaviour that was previously unknown. Here, we present a method to study track annealing *in situ* under high pressure by using synchrotron-based small angle x-ray scattering (SAXS). To simulate fission tracks in a controlled environment, ion tracks were created in apatite from Durango, Mexico using 2 GeV Au or Bi ions provided by an ion accelerator facility. Samples were annealed at 250 °C at approximately 1 GPa pressure using diamond anvil cells (DACs) with heating capabilities. Additional *in situ* annealing experiments at ambient pressure and temperatures between 320 and 390 °C were performed for comparison. At elevated pressure a significantly accelerated annealing rate of the tracks was observed compared with annealing at ambient pressure. However, when extrapolated to geologically relevant temperatures and pressures, the effects become very small. The measurement methodology presented provides a new avenue to study materials behaviour in extreme environments.

## Introduction

Ion tracks can occur when swift heavy ions penetrate solids; they typically form narrow, straight damage trails as a result of the interactions of the energetic ions with the electrons of the target material^1,2^. In nature such tracks can occur in minerals, such as mica, apatite and zircon, as a result of spontaneous fission of radioactive uranium impurities present at ppm levels^1^. Natural so called ‘fission tracks’ are comparable in size and structure to ion tracks with a diameter of approximately 10 nm and lengths of several micrometres, as the fundamental mechanisms for their generation are the same as for ion tracks. Ion tracks generated under controlled conditions in large accelerator facilities are thus often used as a proxy to better understand important parameters and relevant properties of fission tracks^3^.

Fission tracks in minerals such as apatite can be used to study the age and thermal history of rocks^1,4,5^. The age is estimated by correlating the areal density of spontaneous tracks revealed by etching to the amount of uranium in the mineral by the well-known decay laws for radioactive material. Determination of the thermal history makes use of the fact that fission tracks shrink over geological timescales in the presence of elevated temperatures. The low temperature thermal history (<~110 °C) can thus be inferred from analysis of the track areal density and track length distribution. Laboratory experiments at higher temperatures as a function of time are used to establish relevant annealing rates that are then extrapolated to geological temperatures and times. Under ambient conditions fission tracks typically only shrink by a small amount over millions of years^6^. Applications of fission track thermochronology include the investigation of the thermal histories of sedimentary basins, as well as studies of tectonic uplift and denudation rates, and dating of volcanic ashes^7^. Even archaeological artefacts can be dated using this technique^1^.

Thermochronology, as well as most fundamental studies on fission tracks, utilize chemical etching that preferentially dissolves the damaged material and enlarges the resulting etch pits from nanometre to micrometre dimensions such that that tracks can be observed under an optical microscope. Kinetic models of annealing derived from laboratory experiments are then used to derive thermal history information from the fission track age and track length distribution of the etched tracks^1,5^. Variations in annealing rates caused by compositional differences of minerals from different locations are taken into consideration from fundamental studies of composition dependent fission track etching^8,9^.

As the minerals are generally exposed to elevated pressures in the Earths’ crust, the influence of pressure on the annealing behaviour of fission tracks has already been discussed since the 1960s. While this effect was generally considered to be negligible^10,11^, a study by Wendt *el al*. has challenged this assumption and suggested slower annealing rates under elevated pressure^12^. These results remained controversial^13,14^ and a more recent investigation by Schmidt *et al*. has found an accelerated annealing behaviour of fission tracks under very high pressures that becomes negligible at geologically relevant values^7^.

Like the majority of studies on fission tracks, these investigations have used chemical etching to follow the recovery process. This requires separate samples for each temperature/pressure point and can thus introduce uncertainties related to the reproducibility of the experimental pressure/annealing and etching conditions as well as variations in the (natural) mineral sample.

In this report, we present a new methodology for studying the annealing of ion tracks under high-pressure *in situ* by means of synchrotron based small angle x-ray scattering (SAXS). The ion tracks consist of amorphous material with a density different from that of the surrounding crystalline matrix^15^. As SAXS is sensitive to density changes on nanometre length scales, ion tracks in apatite can be readily measured using the technique^3,15,16^. Compared to the analysis using chemically etched tracks, where the original track damage is removed, SAXS has the great advantage of providing direct information on the track damage without the use of chemical etching. We have previously demonstrated that SAXS is well suited for the study of ion tracks in minerals with high precision and with sufficient time resolution for *in situ* measurements^3,15^. Annealing under pressure was performed using heatable diamond anvil cells (DACs). The new measurement methodology presented here opens up exciting opportunities for the study of materials under extreme conditions.

## Results and Discussion

**Figure 1:**
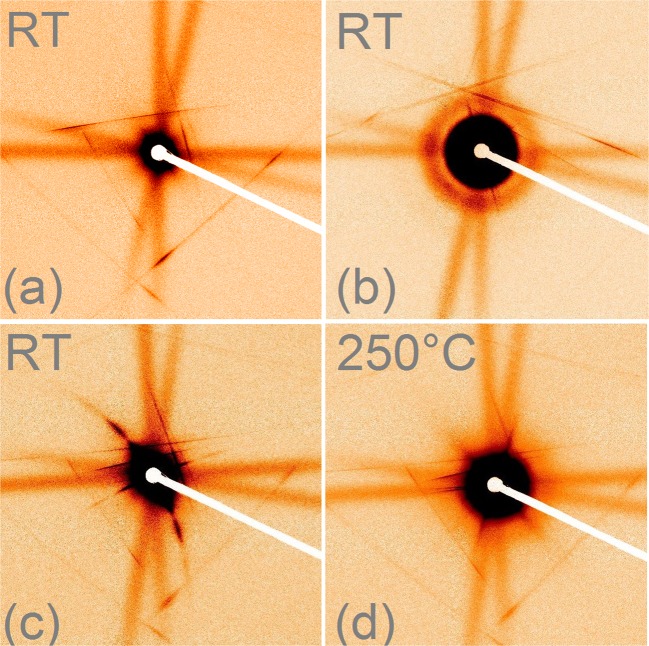
SAXS patterns obtained from virgin and irradiated (2.2 GeV Au ions, 10^11^ cm^−2^) apatite in diamond anvil cells. (**a**) Virgin reference sample without ion tracks showing only the scattering related to the diamond anvils. (**b**) Irradiated sample with the X-ray beam aligned with track direction at RT. (**c**) Irradiated sample with the X-ray beam under a 5° angle with respect to the track axis. (**d**) Irradiated sample after heating at 250 °C for 5 min.

For the experiments we used natural apatite from Durango, Mexico, which is widely used as a standard for fission track dating experiments because it is available as large, gem-quality crystals and has a homogeneous composition. After all natural tracks were annealed from the crystal, new tracks were produced by irradiation with heavy ions at controlled energy and fluence (for details see Materials and Methods). The tracks produced are characterised as long nearly cylindrical zones of amorphous material^15^. Figure 1 shows SAXS patterns obtained from apatite in the diamond anvil cell. In Fig. 1(a) the scattering of a reference sample without tracks is shown. The lines in horizontal and vertical directions result from x-ray scattering in the approximatley 4 mm thick diamonds. The scattering pattern from an apatite sample containing tracks is shown in Fig. 1(b) where the sample is aligned such that the x-ray beam is parallel to the ion tracks. The characteristic scattering from the ion tracks appears as a circular pattern and is a result of the cylindrical cross-section of the parallel oriented ion tracks. When the DAC is tilted by 5° with respect to the incident x-ray beam, the scattering from the tracks becomes anisotropic, changing into slightly curved narrow streaks passing through the beam center (Fig. 1(c)). This results from the large aspect ratios of the tracks that are approximatly 10 nm in diameter but tens of micrometers long^15^. The variation of the scattered intensity along the narrow curved streaks contains information about the radial density profile of the tracks^3,15^. Figure 1(d) shows a scattering image after annealing at 250 °C for the duration of 5 min under a pressure of approximately 1 GPa. The clear weakening of the streak intensity observed from the scattering of the tracks is ascribed to the partial recrystallization of the amorphous ion tracks and the concomitant reduction in the track volume.

**Figure 2:**
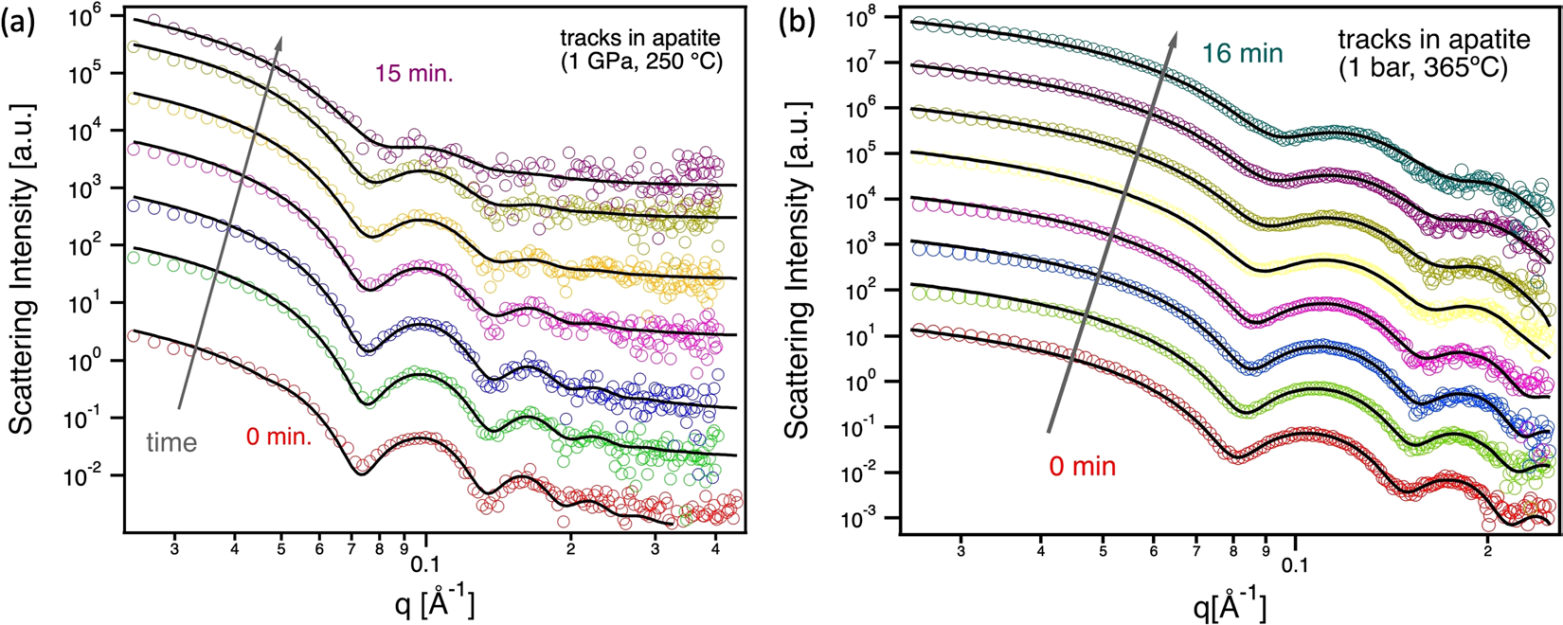
SAXS scattering patters from *in situ* annealing experiments (**a**) at 1 GPa and 250 °C for the first 15 min, and (**b**) at ambient pressure and 365 °C for 16 min.

**Figure 3:**
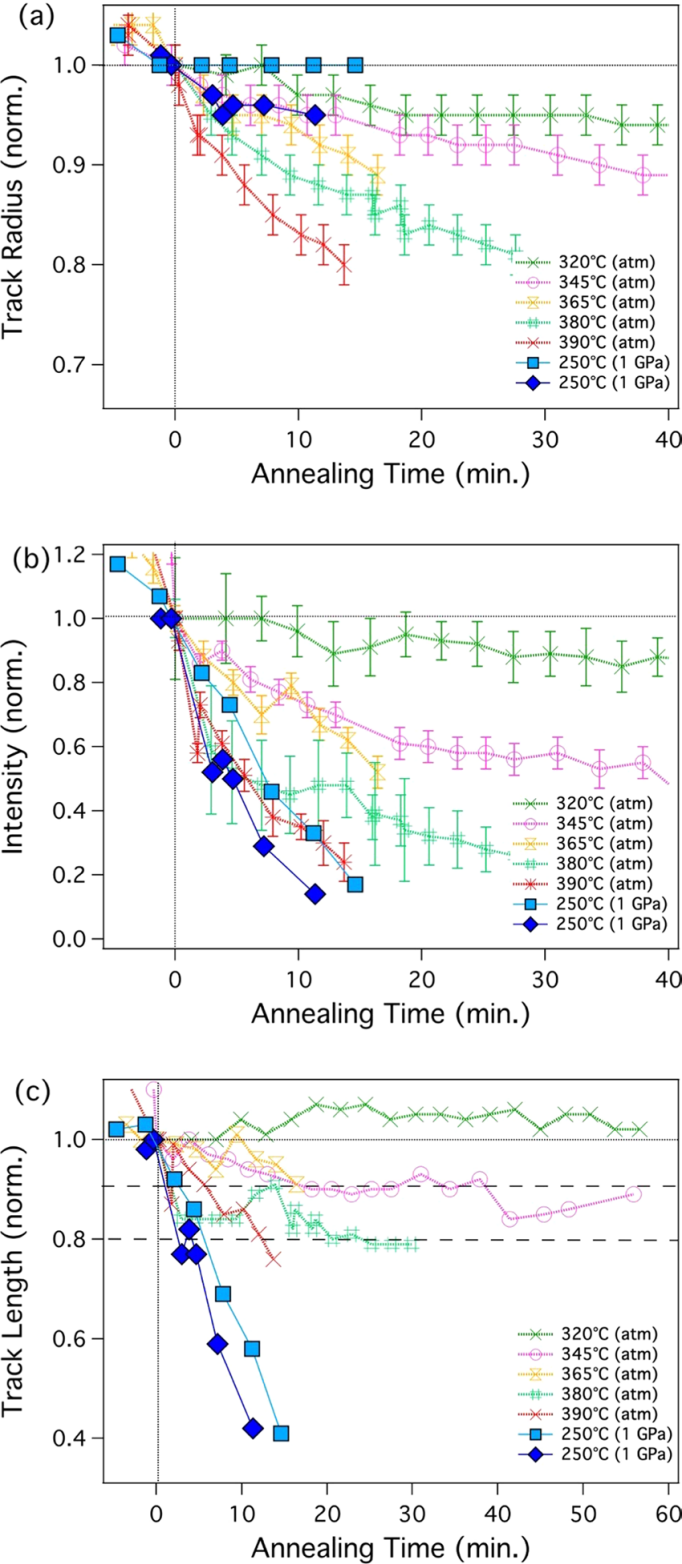
Track parameters as deduced from analysis of the SAXS data as a function of annealing time: normalized track radius (**a**), normalised scattering intensity (**b**), and normalized length (**c**) during annealing (data available as a table in the Supplementary Information).

The scattering intensities along the curved streaks in the scattering images are plotted in Fig. 2 as a function of the scattering vector *q*, which is related to the scattering angle *θ* and the x-ray wavelength λ by the relation $q=\frac{4\pi }{\lambda }\,\sin \,\theta$. The SAXS patterns correspond to isothermal *in situ* annealing runs at a different annealing times under pressure in a DAC at 250 °C (Fig. 2(a)) and at ambient pressure performed in a LINKAM heating stage at 365 °C (Fig. 2(b)). For each pattern the intensity was fitted by modelling the tracks as cylindrical scattering objects with constant density (for details see the Materials and Methods section). This assumption has previously provided a good approximation for the amorphous tracks that occur in apatite^15^. The fits for each pattern were performed using a least square algorithm and are shown as solid lines in Fig. 2. From the fits we can retrieve the radius R of the cylindrical tracks as well as the relative scattering intensity, the latter resulting from the track volume and the density difference between the amorphous track and the crystalline host material $\Delta \rho =\rho -{\rho }_{0}$. Assuming that the density difference $\Delta \rho$ does not change under annealing, the SAXS data allows us to deduce the length of the tracks. We believe this is a reasonable assumption given that the densities of amorphous and crystalline material are generally determined by their phase and there is no indication in the kinetics (see Fig. 3) that multiple processes take place (i.e. track recrystallisation and densification).

Figure 3 shows (a) the normalised track radii *R*, (b) the normalised SAXS intensity *I/I*_0_, and (c) the relative track length *L* derived from the *in situ* annealing measurements under ∼1 GPa pressure (blue squares) and reference samples annealed at ambient pressure (red crosses). The normalised scattering intensity *I/I*_0_ is the intensity for each pattern extrapolated for *q*→0 extracted from the fit divided by the value *I*_0_ prior to annealing. The values were derived from the fits to scattering data shown in Fig. 2. The annealing curves at 250 °C under pressure result from two different annealing series (light blue and dark blue) that were performed to assess reproducibility of the experiments. While the absolute values of the track radii differ somewhat, the general trend is the same, *i.e*., after an initial decrease, the radius remains largely constant at this temperature. The differences between the two runs are mainly attributed to possible small variations in temperature and pressure in the DACs. The uncertainties in the SAXS results associated to the fitting are negligible^15^. With regard to the normalized intensity (Fig. 3(b)) and the related normalized track length (Fig. 3(c)) there is very good agreement between the two runs. We note that SAXS simultaneously measures approximately 10^6^–10^7^ tracks that were produced under identical conditions, yielding good statistics for evaluation of the effects discussed.

Figure 3(b) shows the decrease in normalised scattering intensity *I/I*_0_ for tracks annealed at ambient pressure for constant temperatures of 320, 345, 365, 380 and 390 °C. It is apparent that with increasing temperature the intensity drops at a faster rate. When annealing is performed under hydrostatic pressure of 1 GPa, *I/I*_0_ decreases significantly faster than at ambient pressure, despite the lower temperature of 250 °C. For both high-pressure annealing runs, the decrease in intensity at 250 °C at 1 GPa is similar to that at 390 °C at ambient pressure. This clearly indicates that an increase in pressure results in an accelerated annealing rate.

The SAXS intensity *I* is related to the total volume of scattering objects and the density difference between the scattering objects and embedding matrix material. In our case the former is given by the track volume and the number of tracks, while the latter is given by the difference in density between the tracks and the matrix material $\Delta \rho$. We have assumed that the tracks anneal homogenously at least until the later stages of annealing, where tracks fragment^17^. As such, the number of tracks remains constant at this stage. As previously described, the reduction of the track size can predominantly be described by a recrystallization process^15^, and we assume that the density difference $\Delta \rho$ remains constant. The observed decrease in the scattering intensity can thus be related to the decrease in track volume, which is given by the track radius and length. The radius is determined by the oscillating part of the scattering pattern and is obtained from the fit uncorrelated to the intensity. Figure 3(a) shows the track radius as a function of annealing time for all different temperature and pressure conditions. It is apparent that the samples annealed under high pressure show an initially rapid decrease in track radius within a few minutes after the annealing was started. The decrease is around 0.2–0.4 nm (4–8%) of the initial radius of 5.4 nm, which is similar to that for ambient pressure annealing at 320 °C. After the initial decrease, only small changes can be observed and the track radius remains almost constant for a period of 10–15 min. The SAXS intensity, however, continues to decrease. For annealing at ambient pressure, the tracks show a gradual decrease in radius over the entire annealing time that also appears to be more rapid in the beginning. This two-step annealing has been observed previously and was attributed to different stages of the track recovery process^15^. From the evolution of the track radius, a change in the annealing behavior at elevated pressure cannot be concluded. On the other hand, the decrease in the measured SAXS intensity is clearly significantly enhanced by the presence of pressure. As outlined above, the decrease can be predominantly attributed to a decrease in the track volume. Using the fitted values for radius and intensity we can estimate the normalized track length, which is plotted in Fig. 3(c). This was described in previous work for ion tracks in quartz^16^. The relative track length shows a behaviour comparable to the relative decrease in intensity *I/I*_0_. The decrease under pressure at ~1 GPa at 250 °C is comparable to that at ambient pressure at 390 °C. It is worth noting that we can rule out fragmentation of the tracks into small fragments due to the presence of the streaks in the scattering images that provide evidence for high aspect ratio structures^15,16^. We cannot rule out fragmentation into large segments with aspect ratios of about 1:100. Using transmission electron microscopy, Li *et al*.^17^ have previously shown that under ambient pressure tracks in apatite anneal along the length of the track and fragmentation only occurs at the later stages of annealing.

Our results provide strong evidence that at high pressures the effects of thermal track annealing differ substantially from those at ambient pressure. For pressures around 1 GPa, low temperatures, such as 250 °C, already lead to a significant reduction in the track length, whereas at the same time the track radius decreases only slightly. Quicker annealing along the track length compared to the radial direction was reported earlier^17^, indicating that different annealing processes are operational. Thus, it is not surprising that elevated pressure influences radial and longitudinal track annealing at significantly different rates.

Interestingly, the results are comparable to those from the investigation of fission tracks by Schmidt *et al*.^7^. Using chemical etching and optical microscopy, they also have reported a measurable increase of the track annealing rate at 2 and 4 GPa. However, when they extrapolated the annealing rate to geologically relevant values (<150 MPa), the difference in annealing rate to ambient pressure becomes negligible.

**Figure 4:**
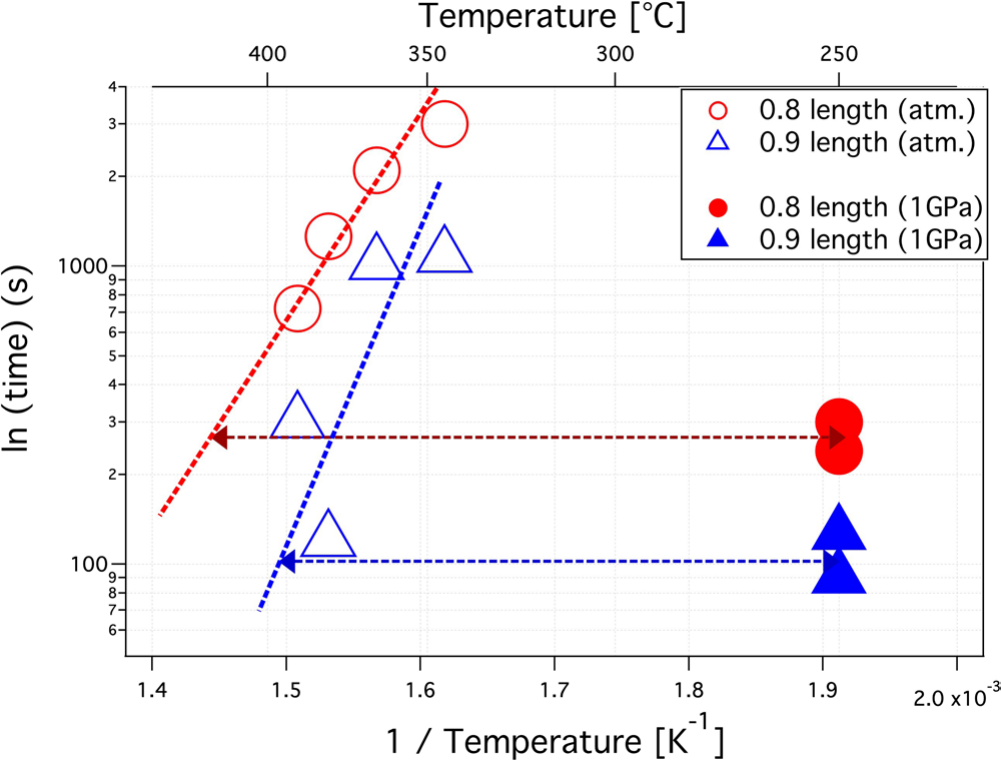
Arrhenius plot of annealing time for reduction of the relative track length to 0.8 (circles) and 0.9 (triangles) as function of reciprocal temperature. For ambient pressure, annealing times for 4 different temperatures are shown (open symbols). For 1 GPa annealing times for 250 °C are shown (solid symbols). The ambient pressure annealing times are extrapolated to the same times as the 1 GPa series. The difference in annealing temperature (double-sided arrows) is used to estimate the influence of pressure on annealing.

We have used the same extrapolation procedure for our data assuming a linear dependence of the track annealing on the reciprocal temperature. For this purpose we estimated the annealing times to reduce the track length to 0.9 and 0.8 from our annealing data indicated by the horizontal dashed lines in Fig. 3c. Figure 4 shows these values in an Arrhenius plot for four annealing temperatures at ambient pressure and the two annealing results at 1 GPa pressure (320 °C annealing at ambient pressure was not used as it did not show a noticeable reduction in track length). In a similar manner as in Schmidt *et al*.^7^, the ambient pressure values are extrapolated to compare these with the 1 GPa samples. As an estimate, to achieve the same track shortening rates as for 1 GPa at 250 °C, temperatures of approximately 145 and 170 °C higher would be required when annealing at 1 atm (based on the 0.8 and 0.9 length retention lines visualised by the double-arrows in Fig. 4). Although this is a significant influence of pressure on track annealing, 1 GPa is significantly higher than the pressures relevant for geological applications. Schmidt *et al*.^7^ estimated the maximum relevant pressure for FT-analysis as 150 MPa for a depth of 5 km at a pressure gradient of 30 MPa/km. Assuming linearity, this would reduce the influence of pressure to a temperature increase in the order of only approximately 24 °C in average. This value is similar to that obtained from the etching experiments of Schmidt *et al*.^7^ who conclude that the influence of pressure is negligable in the context of FT analysis.

## Conclusion

A new method to study ion track annealing in apatite from Durango under high pressure *in situ* using small angle x-ray scattering was presented. This technique is reproducible and directly measures the ion tracks without the need for chemical etching and thus directly accesses the radiation damage resulting from high energy particles. The tracks displayed a significantly faster annealing behaviour at ~1 GPa as compared with ambient pressure at comparable temperatures. When extrapolated to the upper limit of geologically relevant values (150 MPa), however, the effect becomes negligible, in agreement with most studies of fission tracks^13^. For more quantitative evaluation of the effect of pressure on track annealing, measurements at different elevated pressures are desirable.

It remains an open question how the formation of tracks in apatite is influenced by the presence of high-pressure. While controlled experiments to study this are challenging, first experiments of track formation in ZrO_4_ using irradiation in DACs have shown there may indeed be an influence^18^. Performing similar experiments in apatite are feasible and could provide further insights important for the thermochronology community.

The results of our pioneering experiments demonstrate the feasibility of studying ion track annealing under high-pressure *in situ* using SAXS. We have previously demonstrated that SAXS is a powerful tool for the investigation of ion track morphology and annealing behavior in many materials. The addition of high-pressure extends the parameter space and opens up new possibilities for studying materials in extreme environments that can be expected to yield valuable mechanistic insights important in a number of scientific and technological areas.

## Materials and Methods

**Figure 5:**
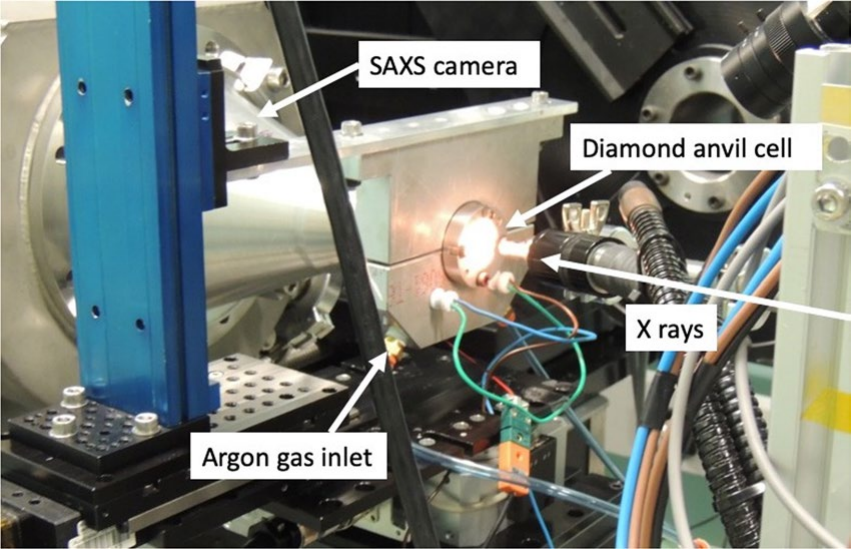
Diamond anvil cell setup at the Australian Synchrotron SAXS/WAXS beamline.

Apatite from Durango, Mexico was first annealed at 400 °C for 24 h to eliminate all natural fission tracks. The apatite crystals were then cut parallel to the c -axis and polished to a thickness of approximately 65 micrometres. To create a uniform ion track distribution, the thin samples were subsequently irradiated with 2.2 GeV Au or 2.3 GeV Bi ions at the UNILAC accelerator at GSI, in Darmstadt, Germany. The irradiations were performed up to a fluence of 1 × 10^11^ ions/cm^2^, under normal beam incidence parallel to the *c*-axis of the crystal^9^. Due to the comparable energy and energy loss values, the Au and Bi ions create tracks of similar size and length. In both cases, the ion range was larger than the sample thickness, which means all tracks are of similar length corresponding to the sample thickness. While the energies used for our irradiation experiments are an order of magnitude higher than those typical for fission fragments, the energy loss is of similar magnitude^3^. Importantly, the fundamental processes for track formation are the same in both cases. The advantages of using high energy ions generated in an accelerator are the nearly uniform energy loss through the samples generating mono-disperse homogeneous nearly cylindrical tracks through the entire thickness of the sample. For the purpose of this study such tracks provide a good model system for fission tracks as small changes can be resolved well using SAXS. Miniaturized pieces (<100 µm in diameter) of the irradiated apatite were pressurized in diamond anvils cells (DACs) with heating capabilities. The sample chamber, consisting of an aperture drilled in a steel gasket with a diameter of ~120 µm, was filled with a 4:1 methanol-ethanol solution to provide hydrostatic pressure conditions. The pressure was controlled by measuring laser-induced fluorescence of a small ruby grain added to the sample chamber^19,20^. In our DACs, pressures between 1 and 50 GPa can be obtained. Elevated temperatures up to 400 °C were achieved by heating coils wrapped around each of the two diamonds. The temperature was controlled by a standard PID controller and measured with a thermocouple that was glued to one of the diamonds. Due to the small sample volume and the high thermal conductivity of the diamond anvils, this results in an accurate temperature measurement and a homogeneous temperature distribution. During heating, the outside of the DAC was purged with argon during the measurements to avoid degradation of the diamonds. A photograph of the DAC mounted at the SAXS/WAXS beamline of the Australian Synchrotron is shown in Fig. 5. For annealing at ambient pressure (1 bar), a LINKAM TS-1500 annealing stage was used. Both, the DAC and the Linkam stage  use resistive heating and a thermocouple positioned very close to the sample. The temperature discrepancy between the LINKAM stage and the DAC is estimated to below 10 °C.

The SAXS experiments were carried out at the SAXS/WAXS beamline at the Australian Synchrotron in Melbourne, Australia^21^. The samples mounted in the DACs were measured in transmission geometry with x-rays of energy 11 keV (λ = 0.1127 nm). Each image was acquired with 20 sec exposure time on a Pilatus 1 M detector. The camera length (sample to detector distance) was accurately calibrated using a silver behenate standard that has well defined sharp scattering rings at known q-values. To analyse the scattering data (see Fig. 2), a model for the track geometry is required. For ion tracks in apatite that consist of amorphous tracks embedded in a crystalline matrix, we have previously demonstrated that a simple cylinder with constant density is an adequate model assumption that yields meaningful and physically sensible results^3,15^. The model describes the scattering intensity $I(q) \sim {|F(q)|}^{2}$ with the scattering amplitude

1 $$F(q)=2\pi {R}^{2}L\Delta \rho \frac{{J}_{1}(q\,R)}{q\,R}$$

for a cylinder with radius *R*, length *L* and density difference to the host material $\Delta \rho =\rho -{\rho }_{0}$.

J_1_ denotes the first order Bessel function. The fit for each pattern was performed using a least square algorithm. The oscillations in the SAXS patterns (see Fig. 2) are related to the track radius while the scattering intensity for q → 0 is a suitable measure of the track fading related to damage recovery as it is proportional to the square root of track area, track length and density change, as shown in Eq. (1) (the oscillating term converges to a constant, independent of R)^16^. Given the radius is known from the fit of the oscillations in the SAXS patterns and assuming the density in the amorphous tracks remains constant, the intensity can directly be related to the track length.

## Supplementary information

Supplementary information

## Data Availability

The datasets generated during and/or analysed during the current study are available from the corresponding author on reasonable request.
